# Lesch-Nyhan Syndrome: Disorder of Self-mutilating Behavior

**DOI:** 10.5005/jp-journals-10005-1350

**Published:** 2016-06-15

**Authors:** Prasad Jathar, Amey M Panse, Madhura Jathar, Pritesh N Gawali

**Affiliations:** 1 Reader, Department of Pedodontics and Preventive Dentistry, Sinhgad Dental College and Hospital, Pune, Maharashtra, India; 2 Senior Lecturer, Department of Pedodontics and Preventive Dentistry, Sinhgad Dental College and Hospital, Pune, Maharashtra, India; 3 Senior Lecturer, Department of Oral Medicine and Radiology, Sinhgad Dental College and Hospital, Pune, Maharashtra, India; 4 Postgraduate Student, Department of Pedodontics and Preventive Dentistry, Sinhgad Dental College and Hospital, Pune, Maharashtra, India

**Keywords:** HPRT enzyme, Lesch-Nyhan syndrome, Self-mutilating behavior.

## Abstract

Lesch-Nyhan syndrome (LNS), a rare inborn error of metabolism, is characterized by self-injurious behavior, which results in partial or total destruction of oral and perioral tissues and/ or fingers. Persistent self-injurious behavior (biting the fingers, hands, lips, and cheeks; banging the head or limbs) is a hallmark of the disease. Prevention of self-mutilation raises significant difficulties. A case of a 10-month-old boy with aggressive behavior and severe lower lip injuries is presented.

**How to cite this article:** Jathar P, Panse AM, Jathar M, Gawali PN. Lesch-Nyhan Syndrome: Disorder of Self-mutilating Behavior. Int J Clin Pediatr Dent 2016;9(2):139-142.

## INTRODUCTION

In the world of developmental and physical disorders, none is stranger than Lesch-Nyhan syndrome (LNS). It is an extremely rare X-linked recessive error of purine metabolism due to severe inborn deficiency of hypoxanthine-guanine phosphoribosyl transferase (HPRT) enzyme.^1^ Lesch-Nyhan syndrome was first described in 1964 by Lesch and Nyhan^2^ as a familial disorder of uric acid metabolism and central nervous system function. Patients are found to have high urinary concentrations of uric acid and increased serum uric acid levels due to the HPRT deficiency. They appear normal when they are born, but by about the third month they are unable to lift their heads or sit up. They display hypotonia, a lack of muscle tone, and many also suffer from dystonia, a lack of motor control. Clinically, LNS is characterized by mental retardation, choreoathe-tosis, spastic cerebral palsy, and aggressive self-mutilating behavior.^3^ A consistent presentation in all cases of LNS is the abnormal compulsion toward self-mutilation usually reported after 1 year of age. This behavior begins as soon as the child’s teeth come in and typically results in parents frantically calling pediatricians asking why their children are trying to eat themselves. Perioral self-mutilating behavior is thought to begin with the eruption of teeth. The behavior continues and results in partial or total destruction of perioral tissue, especially the lower lip. Partial or complete amputation of fingers, toes, and tongue is also common. Lesch-Nyhan syndrome sufferers have been known to stab themselves in their eyes with sharp objects and some have bitten off their tongues.^4^ The prognosis for LNS is very poor. Many patients die in their teens and early twenties and are often very fragile. Lesch-Nyhan syndrome sufferers die from kidney failure in most cases and are also susceptible to infections. Many die suddenly and with no explanation, though recent studies show it may have something to do with a failure in respiration processes.^5^ The estimated prevalence of LNS is 1 in 380,000 live births in Canada and 1 in 235,000 live births in Spain,^6^ and it has been rarely reported from India.

This report presents the self-mutilating behavior of a one-and-a-half-year-old child diagnosed with LNS.

## CASE REPORT

**Fig. 1:**
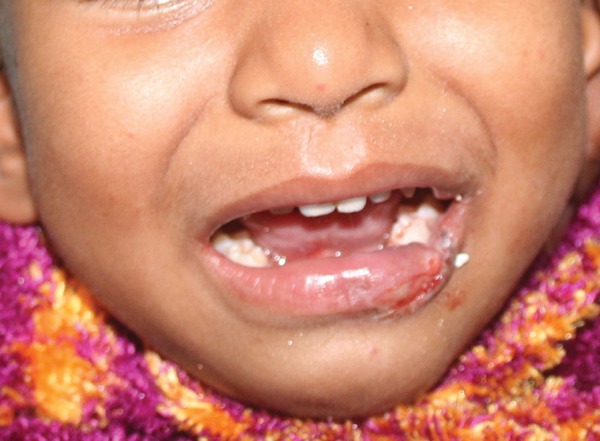
Patient’s extraoral appearance

**Fig. 2:**
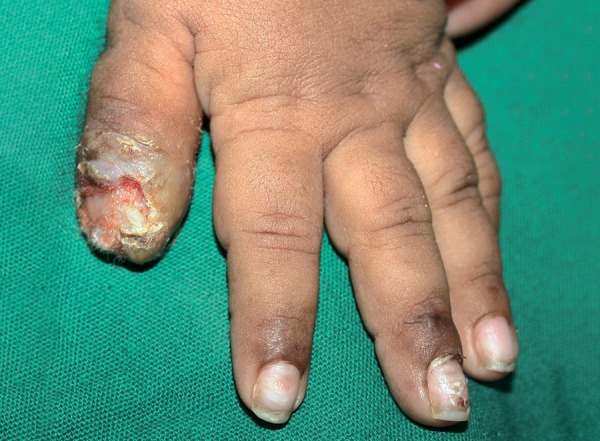
Mutilated thumb of left hand

**Fig. 3:**
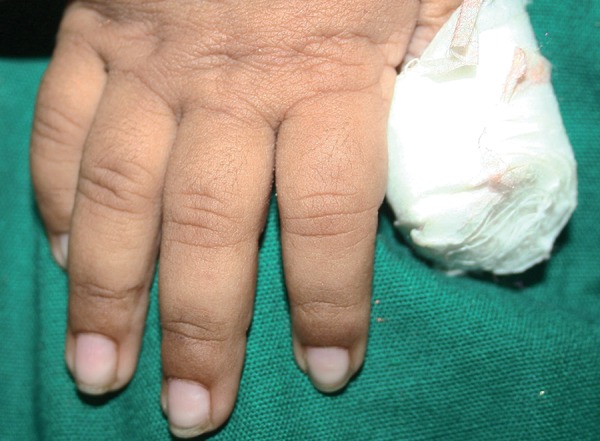
Bandaged thumb on right hand

**Fig. 4:**
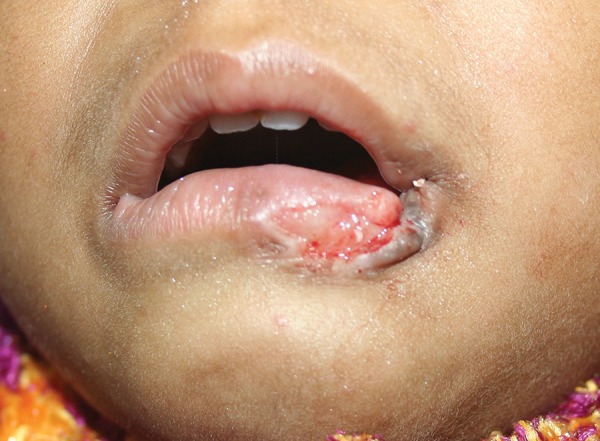
Severely injured lower lip

A one-and-a-half-year-old male child born to a con-sanguineously married couple was brought to the Department of Pedodontics, Sinhgad Dental College, for dermatological assessment of failure to thrive and lacerations over lower lip, thumbs, and index finger due to self-biting (Fig. 1). After recording the history from the parents, it was revealed that the child had developed the habit of self-biting at 10 months of age roughly coinciding with eruption of teeth. He was the only child born to the parents after a normal gestation/pregnancy. He was unable to hold his head yet. He measured 58 cm (50th centile 74.6 cm) in height, weighed 6.3 kg (50th centile 9.08 kg), and had occipitofrontal circumference of 44.9 cm (50th centile 45.3 cm), suggestive of severe growth retardation. His bone age was consistent with his chronological age and mental age was 8 months. On examination, well-defined ulcers/lacerations with crusting and scarring at places involving the left thumb and left index finger were observed (Fig. 2). Also a gauze piece was seen rapped around the child’s right thumb, with the parents giving the history of ulcer wound healing spontaneously after bandaging but recurrence within a day after removal (Fig. 3). Nails of the involved fingers were dystrophic. A single, well-defined, deep ulcer with ragged margins and some scarring was present over the lower lip (Fig. 4). Chorea, hyperreflexia, and positive Babinski’s sign were evident on neurological examination. All deep tendon reflexes were brisk and plantar reflex were extensor on either side. The systemic examination and laboratory investigations (blood cell counts, hepatorenal functions, urinalysis, and chest radiograph) were normal. The serum uric acid levels were 6.5 mg/dl (normal 1.7-5.8 mg/dl), and the urine uric acid: Creatinine ratio was 3:4 (normal 2:5-3:5). Due to lack of availability, the HPRT enzyme estimation in erythrocyte lysate or skin fibroblasts was not carried out. The features of self-mutilating behavior, physical and mental retardation, neurological features, and abnormal urine uric acid:creatinine ratio reached toward the diagnosis of LNS. The treatment plan decided to prevent further trauma to the perioral soft tissue including fabrication of a mouth guard. However, due to lack of patient’s cooperation and as patient was from remote village, proper follow-up could not be maintained.

## DISCUSSION

Lesch-Nyhan syndrome is an extremely rare disorder that strikes the sufferer with debilitating motor and cognitive problems, hyperuricemia, and the urge to harm themselves with acts of self-injurious behavior. Though they are born normal, the infant develops extrapyramidal signs (chorea, dystonia) and pyramidal tract signs (hyperreflexia, sustained ankle clonus, positive Babinski sign, scissoring) become evident usually by 8 to 12 months. Nyhan has stated that next to phenylketonuria, LNS is the second most common inborn metabolic disorder.^7^ The syndrome is caused by a complete or partial lack of activity of HPRT. The HPRT deficiency found in the individuals is closely related with high urinary concentrations of uric acid and increased serum uric acid levels. The diagnosis of LNS is mainly clinical and by serum uric acid estimation. Assay for HPRT enzyme in erythrocyte lysate or demonstration of mosaicism in skin fibroblasts will confirm the diagnosis or carrier state, but poor availability and high cost often limit their use in clinical practice.^8^ Prenatal diagnosis is possible by amniocentesis or chorionic villous sampling. Furthermore, this disorder may be associated with deficits in dopaminergic activity in the basal ganglia as the basal ganglion is involved in voluntary motor control, procedural learning, eye movements, and genitive emotional functions.^9^ Dopamine levels in Lesch-Nyhan patients appear to be down 60 to 80%. In adults, a profound loss of striatal dopamine often causes parkinsonism, but in children it most often causes dystonia, sustained muscle contractions causing twisting or repetitive movements, which is common in Lesch-Nyhan patients.^4^ Serotonin, also a neurotransmitter, has been shown to be regulated by dopamine release in presynaptic channels, and depletion of serotonin will result in high-frequency stimulation, which is seen in Lesch-Nyhan patients.^10^

One of the most striking features of LNS is self-mutilative behavior. The compulsive self-injurious behavior is only shown in patients with complete enzyme (HPRT) defect and some never show autodestructive behavior. The self-injurious behavior usually begins with self-biting at 1 year or may be delayed until teens, resulting in loss of tissue from various sites on the body. Their injurious behavior does not stop with themselves. Lesch-Nyhan syndrome sufferers have been known to punch their doctors,^7^ punch their friends, and even roll their wheelchairs out into the middle of traffic as they yell at the cars not to hit them because it’s the LNS that’s making them do it.^4^ From a dental point of view, self-mutilation can result in massive destruction of the lower lip and to a lesser degree of the upper lip as seen in the present case. The patient used his teeth and fingers to mutilate himself. The fingers are often badly chewed, sometimes down to the bone. Subjects may also use fingers to mutilate themselves. The biting pattern can be asymmetric, with preferential mutilation of the left or right side of the body.^11^ Mutilation is not due to lack of sensation, but is more of an obsessive-compulsive behavior, with the child experiencing pain similar to other normal children. Ulcer, scarring, and missing tissue from the middle and lateral aspect of the lower lip were in the present case recorded on the day the patient first arrived in our department. No signs of tongue scarring were observed as the patient did not appear to bite the tongue. Any object introduced into the mouth was also bitten involuntarily.

Various treatment modalities have been planned in the literature for the prevention of the orofacial mutilat-ing behavior. Extraction of either the primary incisors or canines or all the primary teeth has been advocated as a satisfactory solution in young patients, but the problem of subsequent recurrence of lip biting with the eruption of the permanent dentition then needs to be addressed.^12^ Different intraoral appliances have also been designed such as posterior splint used to create an anterior open bite, soft heat-formed splints, lip bumpers, and mouth guards, but only limited success has been achieved.^13,14^ When the above-mentioned appliance therapy fails, extraction of anterior teeth remains an effective treatment modality to prevent effects of self-mutilation. Treatment for self-mutilation of hands, tongue, and lips with repeated botulinum toxin A injections into the bilateral masseters has been tried, which affects both the central and peripheral nervous systems, resulting in reduced self-abusive behavior in LNS patients.^15^ Treatment with gabapentin has been proven more useful in controlling neuropsychiatric symptoms than carbamazepine or sodium valporate.^16^

## CONCLUSION

Cases of LNS have rarely been reported in India. It has a slow progression, but symptoms can be seen relatively soon after birth. From the first appearance of crystals to the slow neurological progression to the self-destructive behavior, nothing is certain. All patients, however, have one thing in common, the lack of HPRT enzyme. Constant care must be given to these patients. There are no standard methods for prevention of self-mutilation in LNS patients. Appropriate preventive methods have to be developed for each individual patient based on close observation. A suitable oral appliance could be tried initially before employing more invasive approaches.
